# A Checklist for the Conduct, Reporting, and Appraisal of Microcosting Studies in Health Care: Protocol Development

**DOI:** 10.2196/resprot.6263

**Published:** 2016-10-05

**Authors:** Jennifer Prah Ruger, Marian Reiff

**Affiliations:** ^1^School of Social Policy & PracticeUniversity of PennsylvaniaPhiladelphia, PAUnited States; ^2^Perelman School of MedicineUniversity of PennsylvaniaPhiladelphia, PAUnited States; ^3^Department of Medical Ethics & Health PolicyUniversity of PennsylvaniaPhiladelphia, PAUnited States

**Keywords:** microcosting, economic evaluation, cost analysis, checklist, guidelines

## Abstract

**Background:**

Microcosting is a cost estimation method that requires the collection of detailed data on resources utilized, and the unit costs of those resources in order to identify actual resource use and economic costs. Microcosting findings reflect the true costs to health care systems and to society, and are able to provide transparent and consistent estimates. Many economic evaluations in health and medicine use charges, prices, or payments as a proxy for cost. However, using charges, prices, or payments rather than the true costs of resources can result in inaccurate estimates. There is currently no existing checklist or guideline for the conduct, reporting, or appraisal of microcosting studies in health care interventions.

**Objective:**

The aim of this study is to create a checklist and guideline for the conduct, reporting, and appraisal of microcosting studies in health care interventions.

**Methods:**

Appropriate potential domains and items will be identified through (1) a systematic review of all published microcosting studies of health and medical interventions, strategies, and programs; (2) review of published checklists and guidelines for economic evaluations of health interventions, and selection of items relevant for microcosting studies; and (3) theoretical analysis of economic concepts relevant for microcosting. Item selection, formulation, and reduction will be conducted by the research team in order to develop an initial pool of items for evaluation by an expert panel comprising individuals with expertise in microcosting and economic evaluation of health interventions. A modified Delphi process will be conducted to achieve consensus on the checklist. A pilot test will be conducted on a selection of the articles selected for the previous systematic review of published microcosting studies.

**Results:**

The project is currently in progress.

**Conclusions:**

Standardization of the methods used to conduct, report or appraise microcosting studies will enhance the consistency, transparency, and comparability of future microcosting studies. This will be the first checklist for microcosting studies to accomplish these goals and will be a timely and important contribution to the health economic and health policy literature. In addition to its usefulness to health economists and researchers, it will also benefit journal editors and decision-makers who require accurate cost estimates to deliver health care.

## Introduction

As health care costs increase worldwide, there is growing pressure to more efficiently use our limited health care resources. Economic evaluations are used to identify, measure, and compare the costs of health interventions and programs in order to help make decisions about resource allocation and program implementation [1]. A first step for controlling costs is the accurate measurement of the true costs of health interventions and programs. Many economic evaluations in health and medicine use charges, prices or payments as a proxy for cost. However, using charges, prices or payments rather than the true costs of resources can result in inaccurate estimates [2,3]. Microcosting is a cost estimation method that provides detailed and accurate cost data by direct enumeration and costing of all the resources used in the provision of an intervention [4,5]. In contrast to gross-costing studies, which use reimbursement amounts or charges or aggregate cost estimates, microcosting requires the collection of detailed data on resources utilized and the unit costs of those resources in order to identify actual resource use and economic costs. Microcosting findings more accurately reflect the true costs of an intervention to health care systems and to society, and are able to provide transparent and consistent estimates. Microcosting has been shown to improve the validity and reliability of total cost estimates for hospital services and for diagnostic or treatment interventions where costs are not available or evolving [6-9]. Microcosting involves the direct measurement of cost by observation and survey and is especially useful for identifying the actual costs of new health interventions or programs, when existing administrative data are not sufficiently sensitive or when there are no established estimates for their aggregate costs [10-12]. There is an increasing need for microcosting in decision making in health policy, and it is important that studies are conducted according to consistent principles and that they are reported in a way that allows for transparency and comparability across studies.

The importance of rigor and transparency in reporting of health economic evaluations has been addressed by systematic reviews of economic evaluation studies and the development of standards and guidelines for the conduct and reporting of economic evaluations of health interventions [11,13-16]. However, the existing guidelines and checklists do not provide sufficient detail for the methods and techniques involved in microcosting studies [6,10,11,13,17-19]. These instruments do not provide a methodological framework and analytic components specific to the inclusion of items to evaluate microcosting studies. The value of checklists for improving the quality of studies and reports in health care has been demonstrated [20,21]. However, there is currently no existing checklist or guideline for the conduct, reporting or appraisal of microcosting studies in health care interventions. We propose to develop a formal checklist, informed by a theoretically- and empirically-based framework, for the conduct, reporting, and appraisal of microcosting studies in health care. The checklist will (1) provide a framework and guidance for the conduct of microcosting studies; (2) assist in the development of manuscripts reporting microcosting studies and reviewing the manuscripts for publication; and (3) lead to more consistency and transparency in conducting and reporting of microcosting studies, allowing for comparison of the studies’ findings. Ultimately, this protocol will lead to the development of a checklist for the conduct, reporting, and appraisal of microcosting studies in health care, improving the quality of these studies.

## Methods

The design of this protocol for the development of a microcosting checklist utilizes recommendations in the Guidance for Developers of Health Research Reporting Guidelines [20,22], and draws on approaches described in published reports of checklist development for reporting and appraisal of economic evaluations of health interventions [13-16,23-29].

The checklist will be developed in the following four stages: (1) identification of appropriate potential domains and items, (2) tem selection, formulation, and reduction, (3) external review (further item reduction and revision), and (4) testing and assessment.

### Identification of Potential Checklist Items

To identify the important domains and items to be considered for inclusion in a standardized conducting and reporting guideline for microcosting studies in health care, the following three methods will be used: (1) systematic review of microcosting studies (Method 1), (2) review of checklists and guidelines for economic evaluations of health interventions, and selection of items relevant for microcosting studies (Method 2), and (3) theoretical analysis of economic concepts relevant for microcosting (Method 3). Triangulation of the three methods will produce a preliminary list of items that will be more comprehensive and inclusive than items identified from any one method alone.

#### Method 1

A systematic review is being conducted of all published microcosting studies of health and medical interventions, strategies, and programs [19]. A comprehensive database has been created, consisting of all microcosting studies published in English. A research objective is to evaluate the quality of published microcosting studies in health care. Details of the search criteria and methodology for data extraction are published elsewhere [19].

The research team will critically assess the quality of each microcosting study included in the systematic review using checklists recommended by the Campbell and Cochrane Economic Methods Group [30] for appraising reporting and methodological quality of economic evaluations. Specifically, the Drummond checklist [31] and the Evers checklist [26] will be used to evaluate the quality and risk of bias of single effectiveness studies; the Philips checklist [32] will be used to evaluate the quality and risk of bias of studies that use decision analytic modeling. The Fukuda and Immanaka criteria [16] will be used to assess the transparency of cost estimates. The Consolidated Health Economic Evaluating Reporting Standards (CHEERS) checklist will be used to assess reporting quality [14]. These criteria categorize studies into levels of transparency based on whether the study clarifies the cost components included, reports the quantity and unit price of resources separately, and reports an estimate of each component. These checklists were employed because they provided the most relevant criteria for assessing economic evaluations including costing, even though they were not developed specifically to assess microcosting studies.

The checklist items will be filled out independently by the two reviewers conducting the systematic review. Disagreements will be discussed and resolved by the two reviewers, and a third researcher will be consulted if needed. The strengths, inadequacies, and redundancies of the existing checklists used to assess quality and bias of the microcosting studies in the systematic review will be documented. Experience with using the existing checklists (ie, Drummond, Evers, Philips, Fukuda, and CHEERS) for study quality and risk of bias in economic evaluations in the systematic review will demonstrate which items are relevant to assessing the quality and reporting of microcosting studies and which are not. Items are scored as “yes”, “no”, “not clear” or “not applicable”. Those that are scored as “not applicable” by all three reviewers for all studies will be excluded. We will document which items in the existing checklists successfully identified relevant criteria for microcosting. Only the relevant items will be selected for consideration for a preliminary list of items to be included in the checklist. Some items may be modified to fit the needs of a microcosting evaluation. We will also note whether the checklists lacked items to assess specific criteria that are relevant, and should be included, for microcosting studies. Criteria that are inadequately covered will be identified and new items will be formulated for these criteria in the new checklist.

The research team conducting the systematic review will extract data from the microcosting studies using a standardized data collection form based on the CHEERS guidelines [14,15], guidance from the Campbell and Cochrane Economics Methods Group [30], and the research team's previous experience with systematic reviews of health economic studies and microcosting studies [33-38]. Data will be extracted from microcosting studies in a number of areas including (1) cost components included (eg, personnel costs, consumables/materials/supplies cost, medication costs, facility costs, transportation costs, productivity loss); (2) whether the study reports input utilization quantity and unit cost data separately; (3) method of quantity data collection used (eg, time-motion study, patient self-report, cost-accounting database, provider/staff interview); and (4) method of unit cost data collection (eg, invoice amount, hospital/clinic/provider price catalogue, standard fee schedule) [19]. New items will be formulated by the research team conducting the systematic review based on the data extracted from and critical review of the published microcosting studies.

#### Method 2

A comprehensive search for published checklists and guidelines used to evaluate the quality, conduct, and reporting of costing in economic analyses of health interventions and programs will be performed. References to published articles describing reporting guidelines or checklists to evaluate the quality of economic evaluations of health interventions will be identified. The references in the selected articles will also be manually reviewed in an iterative process to identify all relevant checklists and guidelines. We will also manually review the references from systematic reviews conducted to evaluate the conduct and reporting of economic evaluations of health interventions including the CHEERS statement [14,15], and the “Best practices for conducting economic evaluations in health care: a systematic review of quality assessment tools” AHRQ report [13].

The search will include the articles in the systematic review (Method 1). In addition, the terms used to index the relevant articles will be identified and used to perform a broad electronic literature search to identify additional checklists and guidelines. Searches based on terms identified to date include (1) (“microcost” OR “microcost”) AND (“questionnaire” OR “checklist” OR “guideline”); and (2) (“cost” OR “cost analysis”) AND (“questionnaire” OR “checklist” OR “guideline”) AND “health care quality, access and evaluation”). We will search PubMed, EconLit, BIOSIS Previews, Embase, Scopus and the National Health Service Economic Evaluation database (NHS EED) to identify relevant English language articles.

From the checklists and guidelines identified in the selected publications, the items relevant for assessing or reporting costing of health interventions or programs will be extracted. These items will be compiled into a comprehensive list and categorized into domains. Within each domain, we will review and narrow down selection of items and will remove any duplicates. Only items considered relevant to microcosting will be retained based on consensus of the research team. We will provide the rationale for inclusion or exclusion of each item and domain.

#### Method 3

A theoretical analysis of economic concepts relevant for microcosting will be conducted. A search has been done for literature in welfare economics and microeconomics and for literature in costing that defines microcosting and differentiates microcosting from gross costing and other costing methods. The latter search included articles in the systematic review, references from these articles, and references from checklists for economic evaluations. The difference between the use of charges, prices, or payments to assess costs and estimates of the real costs of resources will be examined. An analytical framework for conducting microcosting studies will be developed and conceptual domains relevant for microcosting will be discussed. Any domains that are missing or not adequately represented in any current economic evaluation checklist will be identified. Newly formulated checklist items (not included in existing checklists) will be developed for each conceptual domain for inclusion in the new checklist.

### Creation of an Initial Item Pool

Items derived using each method (ie, the systematic review, checklist review, and theoretical analysis) will be compiled into a comprehensive list. The overlap and variation in domains and items will be documented and any duplicate or redundant items will be removed. The pool of remaining items will be discussed and evaluated by research team members.

New items may be formulated based on the findings of criteria deemed relevant and necessary for microcosting studies but inadequately covered by existing checklists or guidelines. The addition of new items will also be guided by the analysis of the systematic review and by the theoretical analysis. Through deliberation and discussion, items will be refined and a consensus will be reached in the selection of items to be included in a preliminary list.

### External Review by Expert Panel

The checklist will be developed using a modified Delphi method, designed for reaching consensus among an expert panel. Delphi is a “method for structuring a group communication process” [39]. It consists of an iterative multistage process with the goal of consensus among a group of experts [40,41]. In conventional Delphi exercises a list of issues to be considered is usually developed by open-ended questions in the first round, and the participants usually remain anonymous. The following two major modifications that have been reported in the literature will be employed: (1) the use of a literature review to determine in advance the list of issues to be considered and ranked by the panel [40-42], and (2) the possible addition of an online/electronic panel discussion or workshop following the survey rounds to resolve any lack of consensus [40,43,44]. Usually not all panel participants are able to attend the online/electronic panel or workshop, and the discussion will breach the anonymity of participants who attend. If a panel or workshop is held, permission will be obtained from participants to use their names in any acknowledgements in subsequent publications. These modifications to the traditional Delphi process can save time and financial resources, and are appropriate for our topic, which is limited in scope and requires a narrow range of expertise for which there are only a limited number of qualified panel members [40,43]. Our Delphi process will be similar to that described by Husereau et al in the development of the CHEERS report [15].

An international expert panel will be recruited and a modified Delphi exercise will be conducted to rank the items in the preliminary list. Panel participants will be selected based on their expertise in the conduct and reporting of economic evaluations, and specific expertise in microcosting studies and methodology. Potential panel participants will include (1) content specialists who have conducted and published full microcosting studies and who are identified in the course of the systematic literature review; (2) international researchers who have expertise in economic evaluations for health interventions with specific interest in costing studies; (3) journal editors interested in publishing microcosting studies; and (4) methodologists with expertise in checklist development. Invitation emails will be sent to potential members of the expert panel, including a description of the project and the expected timeline. We will invite participants to complete an initial survey to rank the items and provide comments, and if possible, to complete a follow-up survey and discussion and/or workshop electronically, with results reported according to the Checklist for Reporting Results of Internet E-Surveys (CHERRIES) [45].

The preliminary list of items will be sent to panel participants in the form of a survey. The procedures used in the development of the CHEERS checklist will be followed to obtain feedback from the panel participants [15]. Each panel participant will be asked to rate the importance of each item by using a 10-point Likert scale from 1 (“not at all important”) to 10 (“extremely important”). In addition, they will rate their confidence in judging the importance of each item on the basis of their current knowledge from 1 (“not confident”) to 3 (“very confident”). The participants will also be asked to comment on the wording and options for scoring, and recommend deletion or addition of items. The survey will be accessible either online or in print depending on the preference of the respondent.

Survey responses from round 1 will be recorded in an electronic spreadsheet. The items will be ranked by importance scores weighted by confidence ratings. Categories of importance will be created based on previous published reports [15,22]. Items will be labeled according to their weighted average score. There are various approaches to items rank ordering. We will pilot test the method where items with a weighted average score of more than 8 will be labeled as “very important/included”, 7-8 as “high importance/likely included”, 5-6 as “moderate importance/possibly included”, and 0-4 as “low importance/not included”. Comments for each item will be collated and summarized. The research team will review and revise the item list through discussion based on survey responses.

A revised list of items will be compiled including the information about item ranks and averages, and sent to members of the expert panel who agreed to participate in a second round of review. Respondents will be informed that items with a score of 6 or less (labeled “possibly included”) will be included in the final checklist only if they receive a higher score. Item-specific comments from round 1 will be included below each ranked item. After round 2, items with a score of 6 or less will be labeled “rejected” and not considered for the final checklist [15]. Responses will be categorized as for round 1, and comments will be collated and reviewed by the research team. An online meeting may be convened for expert panel participants to discuss the remaining items. The research team will revise the checklist based on the comments and discussion in the meeting.

### Pilot Test

A selection of the full and predominant microcosting articles from the systematic review data will be used to pilot test the new checklist. The articles will be rated by two independent reviewers. Reliability estimates will be calculated and discrepancies will be discussed with the research team. Items may be modified or further explained in order to improve clarity and comprehension. The checklist scores will also be compared with scores for the coded checklists used in the systematic review (eg, the Drummond, Evers, Philips, Fukuda, and CHEERS checklists) in order to assess external validity.

## Results

The project is currently in progress.

## Discussion

### Principal Findings

Currently, health systems in the United States and internationally are faced with increasing costs and limited resources. Accurate cost assessments are essential in order to plan programs and enact health care policies in a cost-efficient manner. Microcosting involves the direct measurement of cost by observation and survey and is used to find the actual cost of new health interventions and programs, or when existing administrative data are not sufficiently sensitive [10,12]. The concepts and methodologies for microcosting studies have been evolving over the past three decades [2,6,10,11,17-19,34-36,46-57], and an increasing number of studies have utilized microcosting techniques in recent years. However, existing instruments and guidelines for economic evaluations lack the framework and specific components required to guide the conduct and reporting of microcosting studies. The aim of this project is to develop a checklist, based on theoretical and empirical research and expert review, to assist with the conduct and reporting of microcosting studies. Standardization of the methods will enhance the consistency, transparency, and comparability of future microcosting studies.

Our review of guidelines and checklists included those that were intended for the conduct, reporting, and appraisal of economic evaluations for health interventions. In some cases, the specific purpose was stated, for example the stated purpose of the CHEERS guidelines is for reporting (and not for conduct) of studies. Several checklists have been designed specifically for quality assessment (eg, Drummond, Evers, Philips). These checklists consist of items that may be relevant for a combination of conduct, reporting, and appraisal without indicating a specific intention. In some cases, the content of the item may be similar, but the wording may indicate the relevance for conduct or reporting (eg, “resource use included” vs “resource use stated”). Checklists may not separate the quality of reporting from the validity of the design and conduct of a study, and elements of checklists intended for evaluating the quality of reporting may therefore be used as guidance in designing studies [13]. Our checklist is intended to provide a framework to consider when conducting, reporting, and/or appraising a microcosting study. In designing our checklist, we will be attentive to the purpose of each domain and each item, and will indicate the relevance for conduct, reporting and appraisal.

This protocol draws on recommendations for developing reporting guidelines [20,21], and on methodologies for developing published checklists for economic evaluations of health interventions [13-16]. Initial steps will include identifying a need for new guidance through a systematic literature review of microcosting studies, and a literature search to identify items used for costing in checklists for health economic evaluations and theoretical analyses. These three activities will be used to generate a preliminary list of items to include when conducting and reporting microcosting studies. The preliminary list will be disseminated to members of an expert panel, identified by the research team as having particular expertise in microcosting analysis and economic evaluation. Panel members will be asked to rate the importance of each item on a Likert scale and the average scores, weighted by confidence level, will be used to rank items. Items will then be categorized and ranked by the research team, based on scores and comments of panel members. Panel members will participate in a second round of review and possibly an online meeting to discuss items to be included in the final checklist. The checklist will be evaluated through a pilot test conducted by the research team in order to assess reliability and validity. The pilot test will also identify any issues that require clarification and determine how useable the checklist is in the real world [20].

### Conclusion

This will be the first checklist for the conduct and reporting of microcosting studies, and will be a timely and important contribution to the health economic and health policy literature. In addition to its usefulness to health economists and researchers, it will also benefit journal editors and decision-makers who require accurate cost estimates in order to meet the goals of the health system to efficiently deliver health care, including electronic health (eHealth) interventions [58]. This framework will help to standardize the methods of microcosting, thereby allowing for greater transparency and comparability of costs among different health care interventions and programs.

## Abbreviations

CHEERS: Consolidated Health Economic Evaluating Reporting Standards
CHERRIES: Checklist for Reporting Results of Internet E-Surveys
